# Cryptogenic Ischemic Stroke in Migraine: Role of Patent Foramen Ovale

**DOI:** 10.3389/fpain.2022.823595

**Published:** 2022-02-25

**Authors:** Cédric Gollion, Fleur Lerebours, Marianne Barbieux-Guillot, Vincent Fabry, Vincent Larrue

**Affiliations:** ^1^Department of Neurology, University Hospital of Toulouse, Toulouse, France; ^2^ToNIC, Toulouse NeuroImaging Center, University of Toulouse, Inserm, Université Paul Sabatier (UPS), Toulouse, France

**Keywords:** migraine, migraine with aura, stroke, stroke in young adults, cryptogenic ischemic stroke, patent foramen ovale

## Abstract

**Introduction:**

Migraine with aura (MWA) has been associated with cryptogenic ischemic stroke (CIS) after adjustment for the presence of a patent foramen ovale (PFO) assessed by a transcranial Doppler. This study aimed at evaluating the association of MWA with causal PFO assessed by Transesophageal echocardiography (TEE) in CIS.

**Methods:**

Patients aged 18–54 years consecutively treated for first acute ischemic stroke in a university hospital stroke unit, between January 2017 and December 2019, were included in this cross-sectional study. Associations between migraine subtypes and PFO were tested for all PFO, possibly causal PFO (PFO with large shunt and/or atrial septal aneurysm [ASA]), and the probably causal PFO subset (large shunt and/or ASA, plus risk of paradoxical embolism [RoPE] score ≥ 7). We adjusted the association between migraine subtypes and possibly causal PFO, which included the probably causal subset for age, sex, large artery atherosclerosis, and small vessel disease.

**Results:**

A total of two hundred and two patients with CIS were included, of whom 42/202 (20%) had MWA, 32/202 (15%) had migraine without aura, and 128/202 (63%) had no migraine. MWA was associated with possibly causal PFO (OR = 4.0, 95%CI [1.78–9.3], *P* < 0.001) and with probably causal PFO (OR = 5.4, 95%CI [2.37–13], *P* < 0.001). In a multinomial logistic regression analysis, MWA remained associated with possibly causal PFO (OR = 3.24, 95% CI [1.45–7.2], *P* = 0.004).

**Conclusion:**

In a young adult population with CIS, MWA was strongly associated with possibly causal PFO, i.e., with a large shunt or combined with an interatrial septal aneurysm.

## Introduction

Migraine with aura (MWA) is a risk factor for ischemic stroke. The risk is higher in young people and women (1). However, the mechanisms by which MWA might cause ischemic stroke are uncertain. The pathophysiological mechanism underlying migrainous aura is strongly hypothesized to be a cortical spreading depolarization (CSD) that may be triggered by ischemia and ischemic stroke (2, 3). Mice with a familial hemiplegic migraine 1 mutation have increased susceptibility to ischemic depolarizations (4). This leads to the hypothesis that in persons with a genetic predisposition to migraine, microemboli could trigger CSD events with MWA phenotype, i.e., symptomatic aura (5), and, in some cases cause more severe hypoperfusion and actual infarction. This could explain the connection of microemboli to migraine aura and to migrainous infarction, a rare event where cerebral infarction occurs concomitant with aura (6), as well as the more common causes of infarction occurring remote from the migraine aura. However, this hypothesis does not explain the source of microembolism. One potential source of emboli, including microemboli, is patent foramen ovale (PFO), a common congenital cardiac anomaly that has been associated with migraine with aura (7), and migraine aura-related stroke (8). The relationship between PFO and cryptogenic ischemic stroke (CIS) is more widely debated (9–13). Two recent studies showed a relationship of CIS with MWA (9, 10) that was irrespective of PFO. Interestingly, however, one study showed that in CIS, MWA prevalence increased with the increasing magnitude of the right-to-left shunt (RLS) in patients with PFO (9), and the other showed that there was an interaction between RLS and both migraine and MWA (10). Studies on the association of CIS and MWA are few, and those using transcranial doppler for PFO diagnosis cannot distinguish between those likely to cause stroke and those that are benign (14). Therefore, the association of PFO with MWA in patients with CIS requires better evaluation. The objective of the present study is to use a transesophageal echocardiogram to identify and characterize PFO in young adults with CIS and then evaluate the association of high risk, possibly causal PFO and MWA.

## Methods

The design of this cross-sectional study has been previously described (Gollion et al. in press). Briefly, data from patients aged 18–54 consecutively treated for first-ever acute ischemic stroke in our university hospital stroke unit, from January 2017 to December 2019, were retrospectively reviewed using the electronic database of our institution. Patients with cerebral venous thrombosis, subarachnoid hemorrhage with secondary brain ischemia, or transient ischemic attack, as defined by transient neurological dysfunction without evidence of infarction on brain imaging, were excluded. The non-inclusion criteria were death in the acute phase of stroke, persistent severe aphasia, psychiatric or cognitive disorders, language barrier, and missing data.

### Assessment of Migraine

All patients were systematically screened and evaluated by the same headache specialist to determine the diagnosis of MWA and migraine without aura (MWoA) according to the International Classification of Headache Disorders 3rd Edition (ICHD3), using a structured questionnaire (15). Migraine assessment was performed during the initial hospitalization, or, in patients who had recovered from initial aphasia, during the subsequent outpatient visit, ~3 months later. Patients who could not be interviewed for migraine were not included.

### Vascular Risk Factors

Being overweight was defined as body mass index ≥ 25 kg/m2. Hypertension was defined as persistent systolic blood pressure > 130 mm Hg or diastolic blood pressure > 80 mm Hg documented before stroke, or treatment with antihypertensive drugs before stroke (16). Diabetes was defined as a previous or new diagnosis of type 1- or type 2-diabetes according to the 2019 guidelines of the American Diabetes Association (17). Tobacco use was recorded in patients currently smoking. Hyperlipidemia was defined as previously treated hyperlipidemia, elevated low-density lipoprotein cholesterol > 1.6 g/L, or hypertriglyceridemia > 2.0 g/L.

### Stroke Workup

Cerebral infarction was always confirmed by a visible lesion on an MRI or CT scan. The cause of stroke was diagnosed using a progressive diagnostic algorithm (18). The initial workup, performed in all patients, included brain MRI or CT when MRI was contraindicated, ECG, routine blood studies (complete blood cell count, prothrombin time, activated partial thromboplastin time, fibrinogen, D-dimer, C-reactive protein, serum creatinine, glucose, cholesterol, and triglyceride concentrations) and angiography of cerebral and cervical vessels using magnetic resonance and/or CT angiography. We used axial T1-weighted MRI scans with fat saturation for the diagnosis of arterial dissection. Carotid atherosclerosis was further evaluated with a carotid ultrasound duplex. ECG monitoring using inpatient telemetry for 72 h was performed in patients without definite cause of stroke on initial workup. Transesophageal echocardiography (TEE) and transthoracic echocardiography were also performed in patients without definite cause of stroke on initial workup. Searching for atrial fibrillation was completed by a 24-h Holter ECG and, in selected patients, by prolonged non-invasive or invasive ECG monitoring. Further investigations (e.g., testing for acquired or genetic thrombophilia, CSF analysis, searching for active malignancy, testing for genetic leukoencephalopathies) were performed in patients with suggestive medical history or clinical findings and in patients without potential cause of stroke on initial workup and cardiac evaluation.

### Patent Foramen Ovale (PFO) Assessment

Patent foramen ovale (PFO) was assessed by using TEE at rest and during provocative maneuvers using IV injection of agitated saline. Some right-to-left shunt (RLS) were previously screened and quantified by transcranial ultrasounds, but the diagnosis of PFO was always retained with TEE. PFO and atrial septal aneurysm (ASA) were diagnosed using previously published criteria (19, 20). PFO were classified as large (defined as an RLS ≥ 20 microbubbles, or a maximum separation of the septum primum from the septum secundum ≥2 mm), and non-large otherwise (14, 21). The Risk of Paradoxical Embolism (RoPE) score was calculated for each patient with PFO and a high RoPE score was defined as a score ≥ 7 (22). PFO with ASA or large PFO were classified as possibly causal. If in addition, the RoPE score was ≥ 7, the PFO was further classified as probably causal (23).

### Classification of Stroke and Definition of Cryptogenic Ischemic Stroke

All individual data were reviewed by a senior vascular neurologist and the causes of stroke were classified using the ASCOD (A: atherosclerosis; S: small-vessel disease; C: cardiac pathology; O: other causes) classification system (24). A grade of 0 was defined as no cause detected despite a complete workup. A potential cause of stroke was defined as an ASCOD grade 1 cause of stroke, an uncertain cause was defined as an ASCOD grade 2 cause of stroke, and a cause present but with an unlikely link was defined as an ASCOD grade 3 cause of stroke (24). A grade of 9 was defined when the workup is insufficient to grade the disease. According to ASCOD, a PFO with ASA was classified as grade 2 and PFO without ASA a grade 3 cause of stroke (24). Cryptogenic ischemic stroke was defined as no grade 1 cause of stroke.

### Data Analysis

No statistical power calculation was conducted prior to the study and the sample size was based on the available data. Data were described as median and interquartile range for continuous variables, and number and percentage for categorical variables. Univariate analysis was performed using a Wilcoxon test, and the χ^2^ or the Fisher exact test. We used multinomial logistic regression analysis to evaluate the association of migraine with aura and migraine without aura (outcome variable, patients no migraine as reference category) with possibly causal PFO. This association was adjusted for age, sex, and minor lesions of atherosclerosis and small vessel disease (grades 2 and 3 according to ASCOD). The latter two variables were chosen to adjust for other possible causes of stroke, rather than their risk factors. In the logistic regression, atherosclerosis and small vessel disease were considered as binary variables (presence or absence). All tests were two-tailed. The significance was set at *P* < 0.05. Power calculation was performed for the main results. Statistics were performed using SPSS, version 25.0 (IBM Corps., Armonk, NY, USA).

### Ethics

The study conformed to the principles outlined in the Declaration of Helsinki. All patients were informed that the clinical data collected during their hospitalization could be used for research purposes and gave their verbal consent. The study was approved by our Institutional Review Board (internal reference RnIPH 2020-28). As the study was part of routine clinical care, no written consent was required.

### Data Availability

Anonymized data not published within this article will be made available by request from any qualified investigator.

## Results

### Population

A total of 464 patients aged 18–54 years were treated for first-ever ischemic stroke at our institution during the study period. Forty-nine patients were not included. The reasons for non-inclusion were: fatal stroke (9), persistent severe aphasia (14), language barrier (12), cognitive or psychiatric disorder (7), and missing data (7).

**Figure 1:**
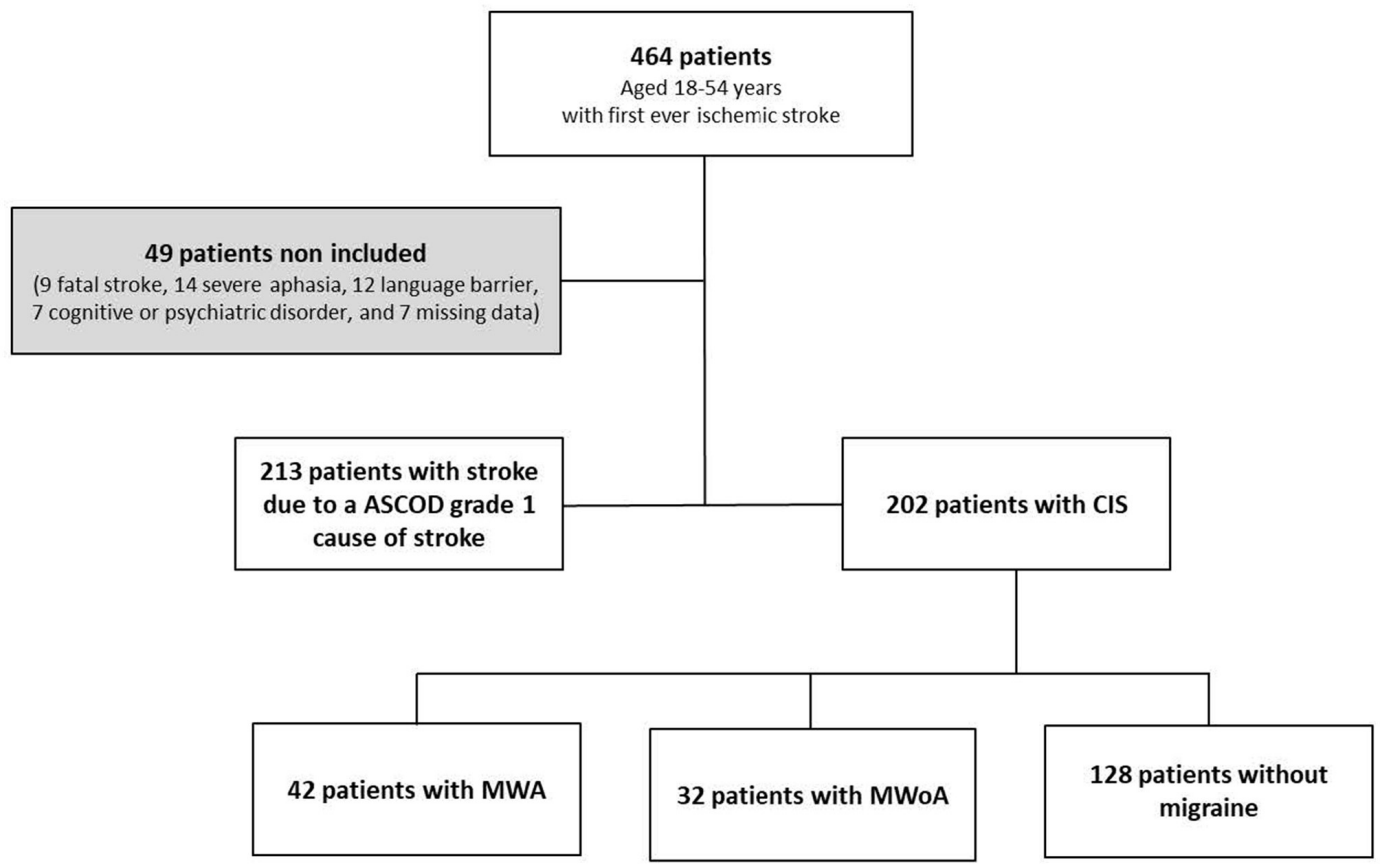
Flow chart of the study. CIS, cryptogenic ischemic stroke; MWoA, migraine without aura; MWA, migraine with aura.

Of the 415 included patients, 202 (48%), had no ASCOD grade 1 cause of stroke and were thus diagnosed with CIS. Among the patients with CIS, 42/202 (20%) had MWA, 32/202 (15%) had migraine without aura (MWoA), and 128/202 (63%) had no migraine (NM). Four MWoA and four MWA patients used triptan for the treatment of migraine. Two MWA patients were on preventive treatment (one aspirin and one amitriptyline). The flow chart is presented in Figure 1. The characteristics of patients with CIS are summarized in Table 1. There were 85 (42%) women and 117 (57%) men with a median age (IQR) of 45 (13.7) years. Patients with MWA were younger, more often women, with less hyperlipidemia and lower body mass index (BMI) than patients without migraine. The rate of minor lesions of atherosclerosis and small vessel disease was lower in patients with MWA than in patients without migraine, respectively OR = 0.342, 95% CI [0.133–0.80], *P* = 0.010 and OR = 0.375, 95% CI [0.106–1.07], *P* = 0.058.

**Table 1:** Characteristics of patients with cryptogenic strokes.

	**No Migraine**	**Migraine**	***P***	**MWoA**	***P***	**MWA**	***P***
	**(*N* = 128)**	**(*N* = 74)**		**(*N* = 32)**		**(*N* = 42)**	
Women (%)	37 (28)	48 (64)	<0.001	24 (75)	<0.001	24 (57)	0.001
Age, median (IQR), years	47 (13)	43 (14)	0.002	43 (13)	0.085	43 (13)	0.004
BMI, median (IQR), kg/m^2^	25.6 (6.2)	23.5 (4.9)	0.002	23.1 (4.1)	0.009	24.0 (5.0)	0.036
Overweight (%)	72 (56)	29 (39)	0.019	11 (34)	0.029	18 (42)	0.153
Hypertension (%)	34 (26)	9 (12)	0.384	4 (12)	0.108	5 (11)	0.057
Diabetes (%)	10 (7.8)	4 (5.4)	0.774	2 (6.2)	> 0.999	2 (4.7)	0.732
Hyperlipidemia (%)	36 (28)	12 (16)	0.060	7 (21)	0.656	5 (11)	0.037
Tobacco use (%)	58 (45)	32 (43)	0.883	18 (56)	0.323	14 (33)	0.209
Use of Oestrogen (%)	6 (16)	12 (25)	0.294	9 (37)	0.064	3 (12)	> 0.999
Atherosclerosis grade 2 and 3	57 (45)	19 (26)	0.010	10 (31)	0.229	9 (21)	0.010
Small vessel disease grade 2 and 3	34 (26)	9 (12)	0.019	4 (12)	0.109	5 (12)	0.058

*P values are given for Migraine, MWoA and MWA vs. No Migraine*.

*MWoA, Migraine Without Aura; MWA, Migraine With Aura; BMI, body mass index; IQR, interquartile range*.

### CIS and Patent Foramen Ovale

The rate of CIS was not significantly higher in patients with MWA (42/76 or 55%) than in patients with MWoA (32/68 or 47%) and patients with NM (128/271 or 47%). The difference between MWA and NM was not significant OR = 1.39, 95%CI [0.81–2.38], *P* = 0.243.

A total of 190/202 (94%) patients with CIS underwent TEE for the assessment of PFO. TEE was not performed in 3/42 (7.1%) MWA, 3/32 (9.3%) MWoA, and 6/128 (4.6%) NM patients. MWA was significantly associated with all PFO, possibly causal PFO, and probably causal PFO. This association was stronger for possibly causal PFO (OR = 4.0, 95%CI [1.78–9.3], *P* < 0.001) and for the probably causal PFO subset (OR = 5.4, 95%CI [2.37–13], *P* < 0.001) than for all PFO (OR = 2.92, 95%CI [1.31–6.7], *P* = 0.005). The power calculations were 0.93, 0.98, and 0.77, respectively. MWOA was not associated with PFO. Possibly causal PFO represented 55% of CIS in MWA patients vs. 34% in MWoA patients and 25% in NM patients (Table 2).

**Table 2:** Association of PFO with MWA and MWoA.

**Types of PFO**	**NM**	**MWA**	**MWoA**	**MWA vs. NM**	**MWoA vs. NM**
	**(*N*=128)**	**(*N* =42)**	**(*N* =32)**		
All PFO *N* (%)	46 (35)	25 (59)	12 (37)	OR = 2.92, 95%CI [1.31–6.7], *P* = 0.005	OR = 1.16, 95%CI [0.46–2.86], *P* = 0.832
Possibly causal PFO *N* (%)	32 (25)	23 (55)	11 (34)	OR = 4.0, 95%CI [1.78–9.3], *P* < 0.001	OR = 1.71, 95%CI [0.66–4.3], *P* = 0.167
Probably causal PFO *N* (%)	23 (17)	22 (52)	9 (28)	OR = 5.4, 95%CI [2.37–13], *P* < 0.001	OR = 1.92, 95%CI [0.68–5.1], *P* = 0.204

*NM, no migraine; MWA, migraine with aura; MWoA, migraine without aura*.

The multinomial logistic regression analysis adjusted for age, sex, and minor lesions of atherosclerosis and small vessel disease showed a strong significant association of possibly causal PFO with MWA (OR = 3.24, 95% CI [1.45–7.2], *P* = 0.004) and no significant association with MWoA (OR = 1.41, 95% CI [0.55–3.63], *P* = 0.470) (Table 3).

**Table 3:** Multinomial logistic regression of the association between migraine without aura and migraine with aura, with possibly causal PFO after adjustment on age, sexe and atherosclerosis, and small vessel disease.

	**Odds ratio (95%CI)**	***P***
No migraine	Reference	
Migraine without aura	1.41 (0.55–3.63)	0.470
Migraine with aura	3.24 (1.45–7.2)	0.004

## Discussion

In this study in young adults with ischemic stroke, we found no significant differences in the rate of CIS according to migraine status. However, in patients with CIS, MWA was associated with PFO showing features suggesting a possibly causal relationship with emboli-related stroke.

Previous studies showing an association of MWA with CIS in young adults compared patients with cryptogenic stroke to healthy controls. This association was independent of the presence of PFO (9, 10). However, MWA is also associated with atrial fibrillation (AF) (6, 25, 26), which may mitigate the association of MWA with CIS when using patients with stroke of known cause as controls. Thus, the Oxford population-based study that compared patients with CIS to patients with stroke of identified cause did not show a higher prevalence of cryptogenic stroke in migraine patients under 55 years. Cryptogenic stroke was more common in elderly migraine patients, but PFO screening was not routinely performed, which may partly explain this association. In addition, as the maximum screening for paroxysmal AF was limited to 5-day ambulatory ECG monitoring, low-burden paroxysmal AF may have been under-detected (13). Further studies using long-duration recordings with insertable cardiac monitors are needed to determine whether the incidence of AF after CIS is higher in patients with MWA than in patients without MWA.

Epidemiological data and the results of randomized controlled trials have been used to identify the characteristics of PFOs that may cause a stroke to distinguish them from benign PFOs unrelated to stroke. New diagnostic criteria have recently been proposed to define causal PFOs (23). According to these new criteria, the causal role of PFO is possible if there is: a large shunt; or a PFO associated with an interatrial septal aneurysm. If, in addition, the RoPE score is ≥7, the causal role becomes probable (27). With these new criteria, we found a significant association between MWA and possibly causal PFO. This association was strong and independent of age, sex, minor atherosclerotic lesions, and small vessel disease as defined by the ASCOD classification. MWoA was not associated with probably causal PFO.

Previous studies showing the association between migraine and PFO in patients with CIS have used transcranial Doppler, which does not diagnose interatrial septal aneurysms (9, 10) or has not distinguished MWA from MWoA (28). Our study shows that a significant and high proportion of ischemic strokes, initially classified as cryptogenic in patients with MWA, are possibly caused by PFO. Thus, showing that PFO with high-risk features for embolization is associated with MWA in persons with CIS, strengthens the hypothesis that emboli-related ischemia is the link between MWA and stroke. Possibly causal PFO explained about half of CIS in patients with MWA vs. one-third in patients with MWoA and one-quarter in NM patients. Nevertheless, in our study, about one-quarter of the strokes in patients with MWA had no identified cause even when accounting for possibly causal PFO. Other mechanisms have been proposed to explain the occurrence of ischemic stroke in patients with MWA, including additional sources of emboli from paroxysmal atrial fibrillation and hypercoagulability, as well as vessel wall changes from endothelial dysfunction, increased susceptibility to and sequelae from cortical depolarization, and altered cerebral autoregulation (1, 3, 6, 29–31). However, the clinical significance of these possible mechanisms is currently elusive.

The strengths of our study include the systematic assessment of migraine status by a headache specialist, the evaluation of PFO with TEE, and a consistently comprehensive diagnostic evaluation. Some limitations should be mentioned, such as the relatively small size of the population of our study and its monocentric recruitment. Risk factors for atherosclerosis and small vessel disease were less common in patients with MWA than in patients with NM. However, we adjusted the association of MWA with causal PFO for minor lesions of atherosclerosis and small vessel disease as defined by the ASCOD classification system. We did not test the multinomial regression on probably causal PFO because of subgroups' sample size of 23 NM, 22 MWA, and 9 MWoA, and because probably causal PFO was defined by the RoPE score which took into account the same factors as those exposed to atherosclerosis and small vessel disease. As long-duration cardiac monitoring using insertable devices was performed in only a small number of patients, the rate of paroxysmal atrial fibrillation may have been underestimated.

## Conclusion

In conclusion, in a young adult population with CIS, MWA was strongly associated with PFO with features heightening the risk of stroke (large shunt and/or interatrial septal aneurysm). These possibly causal PFOs were present in half of the CIS in the MWA subset.

## Data Availability Statement

The raw data supporting the conclusions of this article will be made available by the authors, without undue reservation.

## Ethics Statement

The studies involving human participants were reviewed and approved by University Hospital of Toulouse Institutional Review Board (internal reference RnIPH 2020-28). Written informed consent for participation was not required for this study in accordance with the national legislation and the institutional requirements.

## Author Contributions

CG worked on study design, assessment of migraine, stroke work-up, data analysis, and writing the draft. VL participated in the study design, stroke work-up, and performed the revision of the draft. FL, MB-G, and VF participated in the inclusion of the patients and in the stroke work-up. All authors contributed to the article and approved the submitted version.

## Conflict of Interest

The authors declare that the research was conducted in the absence of any commercial or financial relationships that could be construed as a potential conflict of interest.

## Publisher's Note

All claims expressed in this article are solely those of the authors and do not necessarily represent those of their affiliated organizations, or those of the publisher, the editors and the reviewers. Any product that may be evaluated in this article, or claim that may be made by its manufacturer, is not guaranteed or endorsed by the publisher.
